# Magnetic Resonance Imaging Segmentation on the Basis of Boundary Tracking Algorithm in Lung Cancer Surgery

**DOI:** 10.1155/2021/1368687

**Published:** 2021-11-08

**Authors:** Chengmin Liu, Fulin Ye, Yikai Hu, Shengxin Gao, Yu Lu, Yilong Guo

**Affiliations:** ^1^Thoracic Surgery, Pizhou Hospital of Traditional Chinese Medicine, Pizhou 221300, Jiangsu, China; ^2^Respiratory Medicine, Pizhou Hospital of Traditional Chinese Medicine, Pizhou 221300, Jiangsu, China; ^3^Department of Imaging, Pizhou Hospital of Traditional Chinese Medicine, Pizhou 221300, Jiangsu, China; ^4^Department of Oncology, Pizhou Hospital of Traditional Chinese Medicine, Pizhou 221300, Jiangsu, China

## Abstract

This work was to study the guiding value of magnetic resonance imaging (MRI) based on the target region boundary tracking algorithm in lung cancer surgery. In this study, the traditional boundary tracking algorithm was optimized, and the target neighborhood point boundary tracking method was proposed. The iterative method was used to binarize the lung MRI image, which was applied to the MRI images of 50 lung cancer patients in hospital. The patients were divided into two groups as the progression-free survival (PFS) and overall survival (OS) of surgical treatment group (experimental group, *n* = 25) and nonsurgical treatment group (control group, *n* = 25). The experimental group received surgical resection, while the control group received systemic chemotherapy. The results showed that the traditional boundary tracking algorithm needed to manually rejudge whether the concave and convex parts of the image were missing. The target boundary tracking algorithm can effectively avoid the leakage of concave and convex parts and accurately locate the target image contour, fast operation, without manual intervention. The PFS time of the experimental group (325 days) was significantly higher than that of the control group (186 days) (*P* < 0.05). The OS time of the experimental group (697 days) was significantly higher than that of the control group (428 days) (*P* < 0.05). Fisher exact probability method was used to test the total survival time of patients in the two groups, and the tumor classification and treatment group had significant influence on the OS time (*P* < 0.05). The target boundary tracking algorithm in this study can effectively locate the contour of the target image, and the operation speed was fast. Surgical resection of lung cancer can improve the PFS and OS of patients.

## 1. Introduction

Lung cancer, a malignant tumor, mainly originated from the mucosal epithelium of the bronchus. According to the pathological changes, it can be divided into small cell carcinoma and nonsmall cell carcinoma [1–3]. In recent years, with the serious air pollution and the gradual deterioration of the environment, the mortality rate of global lung cancer is on the rise, which seriously threatens the health and life of the people. According to the National Cancer Center, the number of new lung cancer cases in China reached 816,000 in 2020, accounting for 17.9% of all cancers. On average, 16 people get sick every 10 minutes. The number of deaths was 719,000, accounting for 23.8% of all cancers [4]. Surgical resection is the main way of cancer treatment. According to the degree of tumor resection and residual degree, lung cancer surgery can be divided into uncertain resection, incomplete resection, and complete resection. Uncertain resection is the naked eye can see the tumor remains. Incomplete resection is complete tumor resection under the naked eye, but there are still residues under the light microscope. Complete resection is the complete tumor resection under the naked eye, and there is no residue under the light microscope [5]. Studies reported that the 5-year survival rate of patients with early lung cancer after surgical treatment was 45–65% and that of patients with advanced lung cancer after nonsurgical treatment was 5–10% [6–8]. At present, there is a lack of the fast and accurate lung cancer screening method in clinics, and the early clinical manifestations are not significant. Once it is found to be in the middle and late stages, the best treatment opportunity is missed, which increases the difficulty of clinical treatment [9]. Therefore, a good early diagnosis method is of great significance to improve the clinical efficacy of lung cancer.

The sensitivity of low-dose CT examination in high-risk groups of lung cancer is 5–8 times that of ordinary chest radiography, but it has high radiation hazard and low specificity, which is easy to cause excessive examination [10]. Magnetic resonance imaging (MRI) has a good resolution for soft tissue and can be used for multidirectional and multiparameter imaging, which has great advantages in positioning lung tumor treatment, postoperative follow-up, and evaluating the curative effect. Only using MRI to evaluate lung cancer images cannot diagnose tiny lesions, and its accuracy is low. For MRI analysis of the lung, it is necessary to remove nonlung tissues by image segmentation [11]. The method based on feature extraction proposed by Chen et al. [12] has large amount of calculation, slow operation speed, and long time, which cannot meet the clinical needs. The watershed algorithm proposed by Hu et al. [13] has fast operation speed, but it is prone to excessive segmentation. The boundary tracking algorithm is a key step in target recognition. The boundary tracking algorithm is a key step in target recognition. Given the boundary information of MRI images, features can be extracted according to their characteristics, and then, patterns can be classified and recognized. The lower the boundary error extracted by the boundary tracking algorithm, the more accurate the feature result is and the higher the recognition accuracy is [14]. The application of the boundary tracking algorithm in MRI image segmentation of lung cancer patients can greatly improve the reliability of the feature extraction process and improve the accuracy of lesion recognition.

The innovation of this study is to optimize the traditional boundary tracking algorithm and propose the target neighborhood point boundary tracking method to segment the MRI images of 50 patients with lung cancer surgery, aiming to explore the efficacy evaluation and prognosis after lung cancer surgery.

## 2. Research Methods

### 2.1. Research Objects

In this study, 50 patients with lung cancer in hospital from October 25, 2019, to May 15, 2021, were randomly divided into two groups: surgical treatment group (experimental group, *n* = 25) and nonsurgical treatment group (control group, *n* = 25). The experimental group received surgical resection, while the control group received systemic chemotherapy. There were 23 males and 27 females, with an average age of (45.68 ± 13.48) years old. This experiment was approved by ethics committee of hospital. Patients and their families understood the research and signed the informed consent form.

Inclusion criteria: (1) imaging data showed that there was a mass in the lung of the patient, and no radiotherapy or chemotherapy was performed before operation. (2) No tumor metastasis occurred in the comprehensive examination. (3) No second primary lung tumor occurred. (4) Data on chemoradiotherapy of the patient were complete.

Exclusion criteria: (1) tumor pathological nature is not determined, and staging is not accurate. (2) Pregnancy, lactation women, and minors. (3) Distant metastasis of tumor was found. (4) Severe complications such as hypertension and diabetes.

### 2.2. Treatment Methods

The experimental group underwent surgical resection, tracheal intubation anesthesia, and unilateral lung ventilation. After comprehensive evaluation of the patients, the unnecessary surgical types were adopted, among which 12 cases underwent thoracoscopic lobectomy combined with mediastinal lymph node dissection. Combined lobectomy was performed in five cases. Six patients underwent pneumonectomy combined with mediastinal lymph node dissection. Two cases underwent sleeve resection.

The control group received systemic chemotherapy, chemotherapy 2–5 cycles. The standard platinum-containing scheme was adopted. Among them, 8 cases were treated with vinorelbine plus cisplatin (VC). 12 cases were treated with gemcitabine plus cisplatin (GC). 2 cases were treated with paclitaxel plus cisplatin (PC). One case was treated with docetaxel plus cisplatin (DC). Two cases were treated with pemetrexed plus cisplatin (PC). Conventional fractionated dose was 1.5–2.0 Gy, and total dose was 50–65 Gy.

### 2.3. MRI Examination Method

The magnetic resonance instrument is the German Siemens Avanto 1.5 T superconducting magnetic resonance scanner. The gradient field strength is 40 mT/m and 80 mT/s, the switching rate is 250 mT/m/ms and 150 mT/m/ms, and 8-channel skull phased array coil is adopted. Conventional plain scan transverse axial T1 weighted imaging (T1WI): time of repetition (TR) = 240; time of evaluation (TE) = 2.5 ms. T2 weighted imaging (T2WI): TR = 240; TE = 2.5 ms. The contrast agent is meglumine gadolinium pentanoate injection. The high pressure syringe is used for rapid injection through the elbow vein. The injection rate is 2.5 mL/s, and the dose is 0.2 mL/kg. T1WI transverse axial position after enhancement: TR = 240; TE = 2.5 ms. Sagittal position: TR = 240; TE = 2.5 ms. Coronal position: TR = 240; TE = 2.5 ms. FOV = 22 cm × 22 cm, layer thickness of 4.5 mm, and layer spacing of 1 mm.

### 2.4. Follow-Up Method

Comparing the progression-free survival (PFS) between surgical treatment and nonsurgical treatment, it refers to the time from randomization to the first occurrence of disease progression or death from any cause. Overall survival (OS) refers to the time from randomization to death caused by any reason, one-year survival rate and two-year survival rate. The OS time was counted by telephone follow-up and outpatient follow-up. The follow-up time was 1–6 years. The last follow-up was on April 10, 2021. The PFS was counted by outpatient follow-up and inpatient examination. The follow-up time was 1–6 years. The last follow-up was on April 10, 2021. Patients who are unable to contact or unwilling to provide relevant information and patients with unclear inpatient examination data are defined as loss of follow-up.

### 2.5. Boundary Tracking Algorithm

**Figure 1:**
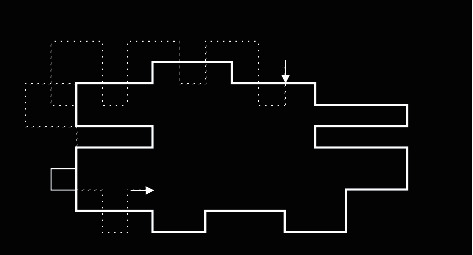
Crawler method determining the target boundary. Note: the arrow indicates the starting point, the solid line indicates the outline of the target object, and the dotted line indicates the action track.

The traditional boundary tracking algorithm uses the crawler method to process data. Any point near the boundary is taken as the starting point, and each step is further taken as a pixel. After stepping from the background area to the target area, each step turns left until it passes through the target area. After stepping from the target area into the background area, each step turns to the right, until it passes through the background area. After a week of circulation around the target, it returns to the starting point. The whole process trajectory is the outline of the target (Figure 1).

**Figure 2:**
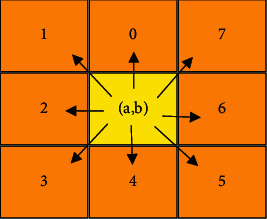
Target points and 8 neighborhoods.

The traditional boundary tracking algorithm can lead to circuitous phenomena in some salient parts of the target. Therefore, this study optimizes the traditional boundary tracking algorithm and proposes the boundary tracking method of target neighborhood points. The object of this algorithm is a binary image, which can select a point at the edge of the target area as the starting point, and then use clockwise search to view the background area as a plane, and the target area is a lung tumor. In digital images, each pixel (a, b) contains eight neighborhoods, so the direction of each boundary point can be extended to eight directions (0, 1, 2, 3, 4, 5, 6, 7), as shown in Figure 2.

**Figure 3:**
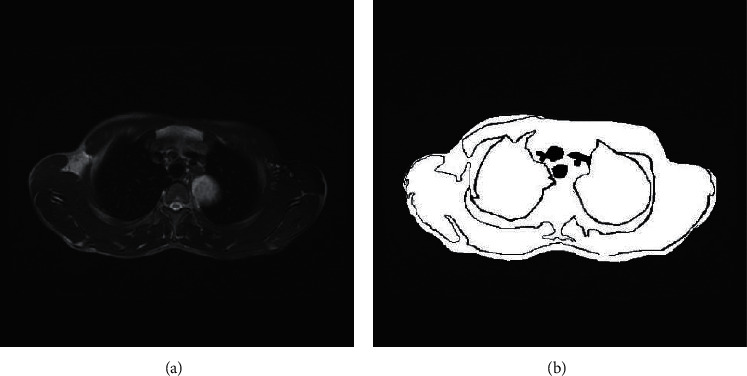
Comparison of MRI images of lung tumors before and after binarization. (a) Original MRI of pulmonary tumors. (b) MRI after lung tumor binarization.

The background color, lung tissue, and nonlung tissue color difference of lung MRI are significant. Transforming the image into binary image can not only improve the operation speed but also reduce the boundary damage of lung MRI. In this study, the iterative method is used to determine the threshold of the image. The pixel gray value below the threshold is set to 0, and the gray value above the threshold is set to 1. The image binarization processing results are shown in Figure 3.

The algorithm of boundary acquisition starts from the starting point and scans the point with a pixel value of 0 line by line and point by point from top to bottom, left to right. If the continuous pixel value of its right neighborhood is 1, the point is determined as the starting point of the boundary. Among the eight neighborhood points around the starting point, the next boundary point is searched according to the proximity principle and pixel principle to determine whether this point is in the array to avoid repeated entry and lead to dead loop. When the starting point of the boundary forms a closed boundary, the boundary points are saved in the array. On the basis of the original image, the region growth method is used to extract the lung tissue image, the center of the original image is constantly radiated to the surrounding, and the gray values of the peripheral pixels are all set to 0. The final image is lung tumor imaging.

### 2.6. Experimental Design

In order to intuitively reflect the advantages of the target boundary tracking algorithm, this study compares the target boundary tracking algorithm with the traditional boundary tracking algorithm through simulation experiments, and the simulation process is realized by Matlab programming on the computer. In the simulation experiment, the original lung tumor MRI image is selected as the standard image, with a size of 125 × 125, gray value 247. In the simulation process, in order to make the image display clearer, the binary image in the target detection result is reversed, 0 is changed to 1, and 1 is changed to 0.

### 2.7. Statistical Methods

In this study, SPSS 22.0 statistical software was used to analyze the result data. The calculated data conforming to the normal distribution was expressed by the mean standard deviation $\left(\bar{x}\pm s\right)$, and the calculated data not conforming to the normal distribution were expressed by the percentage (%). The paired data *t*-test was used. Binary logistic multivariate regression analysis was used to score the tendency matching of the baseline data of the two groups. After matching, the Kaplan–Meier curve was used to describe the patient survival data, and the log-rank test was used to compare the survival rates of the experimental group and the control group. The Cox proportional hazards model was used for multivariate analysis. Fisher exact probability method was used to compare the one-year survival rate and two-year survival rate between the two groups. *P* < 0.05 indicated that the difference was significant.

## 3. Results

### 3.1. Simulation Test Results

**Figure 4:**
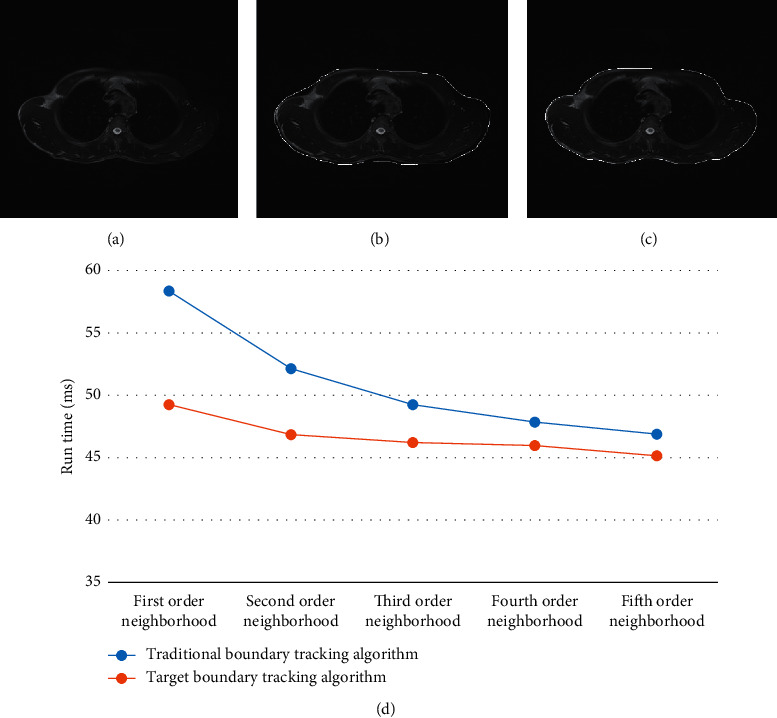
Comparison of lung tissue boundary segmentation. (a) Primitive lung MRI. (b) Segmentation results of the traditional boundary tracking algorithm. (c) Target boundary tracking algorithm. (d) Comparison of different order neighborhood running time between the traditional border tracking algorithm and target border tracking algorithm.

Figure 4 shows the comparison results of lung tissue boundary segmentation between the traditional boundary tracking algorithm and the target boundary tracking algorithm. From the figure, the traditional boundary tracking algorithm needed to estimate the number of repeated tracking in advance. After obtaining the image contour, it needed to carefully judge whether there were omissions in some concave-convex parts manually, and the segmentation effect was not ideal. The target boundary tracking algorithm in this study can effectively avoid the omission of concave-convex parts and can accurately locate the contour of the target image at one time. It had high operation speed and no manual intervention.

It can be seen from Figure 4(d) that the larger the selected neighborhood order, the less time it takes for the boundary tracking algorithm to run. The running time of the target tracking algorithm is lower than that of the traditional border tracking algorithm.

### 3.2. General Information

**Figure 5:**
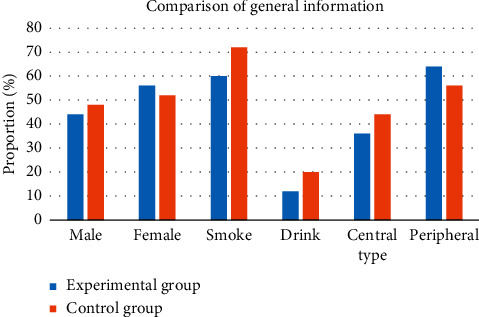
General data statistics.

A total of 50 patients with lung cancer were included in this study, with an average age of (45.68 ± 13.48) years old. In the experimental group, there were 11 males (44%) and 14 females (56%). In the control group, there were 12 males (48%) and 13 females (52%). There were 15 cases (60%) with the smoking history in the experimental group and 18 cases (72%) in the control group, and they were all males. There were 2 cases (12%) with drinking history in the experimental group and 5 cases (20%) in the control group, and they were all males. There is no statistical difference between the two groups (*P* < 0.05), and it is comparable.  Figure 5 shows the detail of general information.

### 3.3. MRI Results

**Figure 6:**
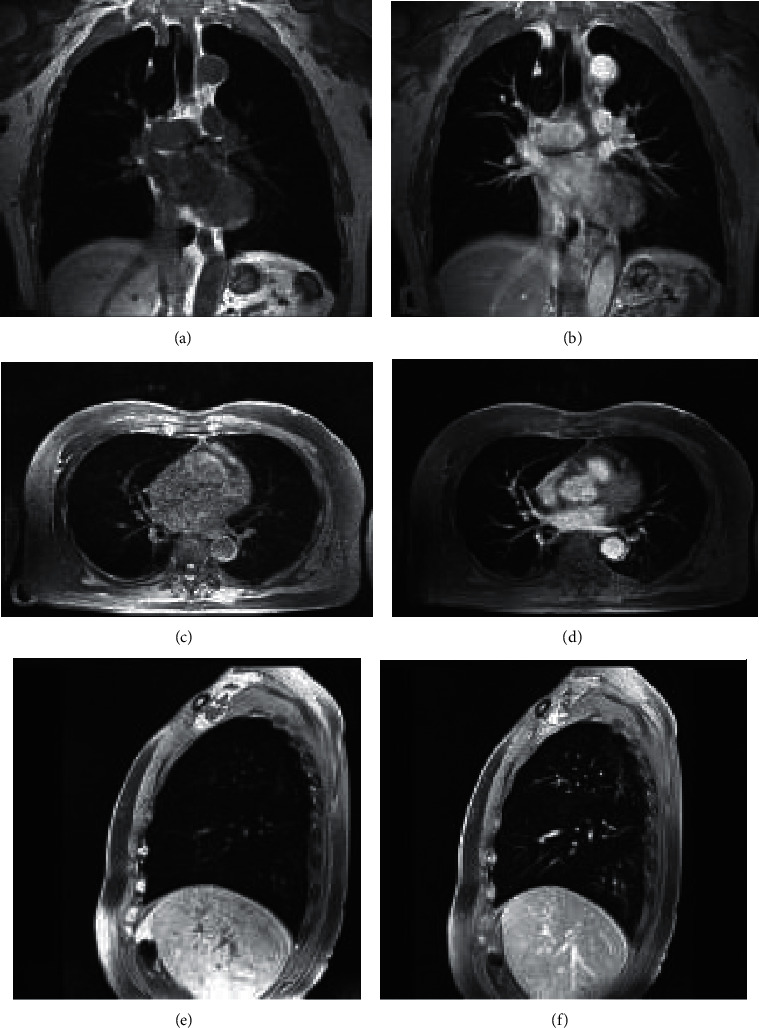
MRI results. (a) Not enhanced coronal. (b) Enhanced coronal. (c) Not enhanced axial. (d) Enhanced axial. (e) Not enhanced sagittal. (f) Enhanced sagittal. Note: male patient's tumors in the posterior segment of the left lung are clearly displayed. Nonvascular branches and bronchi in the lung parenchyma are normally displayed without vascular interference.

Lung cancer mass showed medium uniform signal similar to muscle on T1WI. It was high signal on T2WI, and the signal was mostly uneven. The large mediastinal vessels suggested dark shadow on MRI due to their flow void effect, which was easy to distinguish from tumors. On MRI, there was a layer of high signal fat band around the normal mediastinal large blood vessels, trachea, and bronchus. When the tumor invaded in time, this high signal band disappeared. The inner wall of the contact surface between blood vessels, trachea, and bronchus and the tumor was not smooth, showing thickening and stenosis of the pipe wall. For hilar mediastinal lymph node metastasis, MRI was easy to identify, with medium signal on T1WI and slightly high signal on T2WI.  Figure 6 shows the results of MRI.

### 3.4. Comparison of Survival

**Figure 7:**
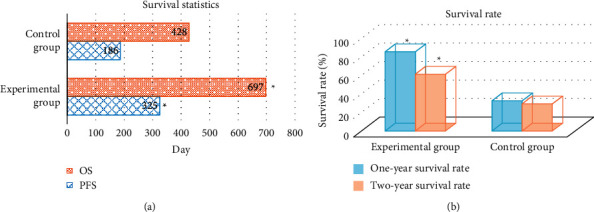
Comparison of survival between the two groups. (a) Comparison of progression-free survival. (b) Survival rate comparison. ^*∗*^Significant difference compared with the control group (*P* < 0.05).

The one-year survival rate was calculated, and the number of patients who survived after 365 days was taken. The two-year survival rate was calculated, and the number of patients who survived after 730 days was taken. The PFS in the experimental group was significantly higher than that in the control group (325 days vs. 186 days) (*P* < 0.05). The OS time of the experimental group was significantly higher than that of the control group (697 days vs. 428 days) (*P* < 0.05). The one-year survival rate of the experimental group was significantly higher than that of the control group (85.7% vs. 32.5%) (*P* < 0.05). The two-year survival rate of the experimental group was significantly higher than that of the control group (61.4% vs. 29.6%) (*P* < 0.05) (Figure 7).

### 3.5. Comparison of PFS

**Figure 8:**
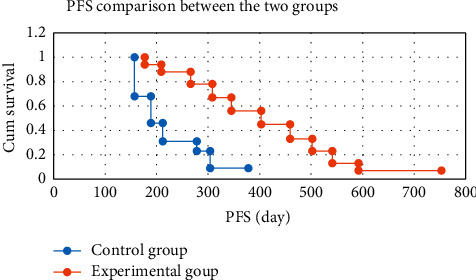
Comparison of progression-free survival between the two groups.

Kaplan–Meier survival analysis was used to analyze the PFS of patients in the two groups. The results showed that the PFS of the experimental group was significantly higher than that of the control group (*P* < 0.05), as shown in Figure 8. According to the clinical expertise, the tumor grading and treatment grouping after screening can be included in the Cox proportional hazards regression model for survival analysis. The results revealed that the effect of tumor grading on PFS was not statistically significant (*P*=0.065 > 0.05). The effect of treatment grouping on PFS was statistically significant (*P*=0.037 < 0.05) (Table 1).

### 3.6. Comparison of Overall Survival

**Figure 9:**
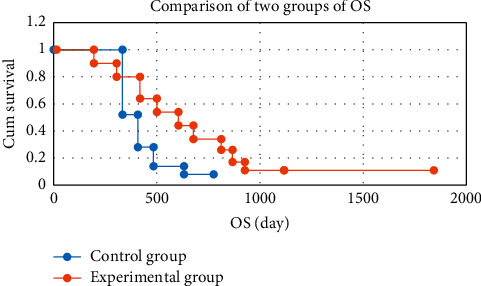
Comparison of overall survival between the two groups.

Fisher exact probability method was used to test OS of the two groups. The results showed that the one-year survival rate and two-year survival rate of the two groups were statistically significant (*P* < 0.05), as shown in Figure 9. According to the clinical expertise, the tumor classification and treatment grouping after screening can be included in the Cox proportional hazards regression model for survival analysis. The results indicated that the impact of tumor classification on OS was statistically significant (*P*=0.034 < 0.05). The effect of treatment grouping on the OS time was statistically significant (*P*=0.045 < 0.05) (Table 2).

## 4. Discussion

Lung cancer is the most common malignant tumor in China, with high morbidity and mortality. If it can be found and diagnosed early, the survival rate of patients can be improved. Efficient detection methods are of great significance for the risk assessment of lung cancer [15–17]. For lung cancer screening and qualitative auxiliary diagnosis software emerge in endlessly, especially the use of different model algorithms, the sensitivity and specificity of the final detection results cannot be unified. In this study, the traditional boundary tracking algorithm is optimized, and a target neighborhood point boundary tracking algorithm is proposed [18]. The traditional boundary tracking algorithm needs to estimate the number of repeated tracking in advance. After obtaining the image contour, it needs to carefully judge whether some concave-convex parts are missing, and the segmentation effect is not ideal. The target boundary tracking algorithm in this study can effectively avoid the omission of concave-convex parts and can accurately locate the contour of the target image at one time. It has high operation speed and no manual intervention. This shows that the target boundary tracking algorithm analyzes the boundary characteristics of lung tissue, optimizes the traditional boundary tracking algorithm, and achieves good segmentation results.

The sample size of this study is small, a variety of influencing factors need to be considered, and the causal relationship is complex. The nonparametric test can be used to create random test conditions. By matching gender, age, tumor grade, treatment grouping, and other factors, the results are statistically more reliable [19, 20]. PFS can quantitatively reflect the growth of tumor and is not affected by postoperative intervention [21–23]. The prolongation of PFS can prolong the tumor remission time and improve the clinical symptoms of patients. The PFS (325 days) in the experimental group was significantly higher than that in the control group (186 days) (*P* < 0.05). The OS time of the experimental group (697 days) was significantly higher than that of the control group (428 days) (*P* < 0.05). Kaplan–Meier survival analysis was used to analyze the PFS of patients in the two groups. The results showed that the PFS of the experimental group was significantly higher than that of the control group (*P* < 0.05), which was consistent with the conclusion of Pezzi et al. [24] that it had a positive impact on the prognosis of patients with small cell lung cancer. The effect of treatment grouping on PFS was statistically significant (*P*=0.037 < 0.05), indicating that treatment grouping was an independent factor affecting the prognosis of patients.

The one-year survival rate of the experimental group (85.7%) was significantly higher than that of the control group (32.5%) (*P* < 0.05). The two-year survival rate of the experimental group (61.4%) was significantly higher than that of the control group (29.6%) (*P* < 0.05). Taunk et al. [25] reported that the one-year survival rate of patients with small cell lung cancer treated surgically reached 91.2%. The low one-year survival rate in this study may be due to the small sample size, vulnerable to individual patients, and many lost visits, which interfered with the results. Moreover, there were few patients under the age of 35 in this study, which also reflected that age had a certain impact on the prognosis.

## 5. Conclusion

In this study, the traditional boundary tracking algorithm is optimized, and the target neighborhood point boundary tracking algorithm is proposed and applied to the MRI images of 50 patients with lung cancer. The target boundary tracking algorithm in this study can effectively avoid the omission of concave-convex parts and can accurately locate the contour of the target image at one time, with high operation speed and no manual intervention. Surgical resection of lung cancer can improve the PFS and OS of patients. The limitation of this study is that the sample size is small, the follow-up time is insufficient, and the number of lost visits is large. In the later stage, we need to enlarge the sample size and try to find out the influence of lung cancer surgery on the five-year survival rate of patients. In conclusion, this study provides a reference basis for clinical treatment of lung cancer, so as to improve the clinical efficacy and prognosis.

## Figures and Tables

**Table 1:** Cox proportional hazards model analysis.

Covariant	Regression coefficient	Wald	95% CI	*P*
Tumor grade	−0.258	3.257	0.528–1.359	0.065
Treatment grouping	0.364	4.332	1.327–1.847	0.037

**Table 2:** Cox proportional hazards model analysis.

Covariant	Regression coefficient	Wald	95% CI	*P*
Tumor grade	−0.475	8.367	0.425–0.853	0.034
Treatment grouping	0.324	4.264	1.356–1.925	0.045

## Data Availability

The data used to support the findings of this study are available from the corresponding author upon request.
